# The FAIR Guiding Principles for scientific data management and stewardship

**DOI:** 10.1038/sdata.2016.18

**Published:** 2016-03-15

**Authors:** Mark D. Wilkinson, Michel Dumontier, IJsbrand Jan Aalbersberg, Gabrielle Appleton, Myles Axton, Arie Baak, Niklas Blomberg, Jan-Willem Boiten, Luiz Bonino da Silva Santos, Philip E. Bourne, Jildau Bouwman, Anthony J. Brookes, Tim Clark, Mercè Crosas, Ingrid Dillo, Olivier Dumon, Scott Edmunds, Chris T. Evelo, Richard Finkers, Alejandra Gonzalez-Beltran, Alasdair J.G. Gray, Paul Groth, Carole Goble, Jeffrey S. Grethe, Jaap Heringa, Peter A.C ’t Hoen, Rob Hooft, Tobias Kuhn, Ruben Kok, Joost Kok, Scott J. Lusher, Maryann E. Martone, Albert Mons, Abel L. Packer, Bengt Persson, Philippe Rocca-Serra, Marco Roos, Rene van Schaik, Susanna-Assunta Sansone, Erik Schultes, Thierry Sengstag, Ted Slater, George Strawn, Morris A. Swertz, Mark Thompson, Johan van der Lei, Erik van Mulligen, Jan Velterop, Andra Waagmeester, Peter Wittenburg, Katherine Wolstencroft, Jun Zhao, Barend Mons

**Affiliations:** 1 Center for Plant Biotechnology and Genomics, Universidad Politécnica de Madrid, Madrid 28223, Spain; 2 Stanford University, Stanford 94305-5411, USA; 3 Elsevier, Amsterdam 1043 NX, The Netherlands; 4 Nature Genetics, New York 10004-1562, USA; 5 Euretos and Phortos Consultants, Rotterdam 2741 CA, The Netherlands; 6 ELIXIR, Wellcome Genome Campus, Hinxton CB10 1SA, UK; 7 Lygature, Eindhoven 5656 AG, The Netherlands; 8 Vrije Universiteit Amsterdam, Dutch Techcenter for Life Sciences, Amsterdam 1081 HV, The Netherlands; 9 Office of the Director, National Institutes of Health, Rockville 20892, USA; 10 TNO, Zeist 3700 AJ, The Netherlands; 11 Department of Genetics, University of Leicester, Leicester LE1 7RH, UK; 12 Harvard Medical School, Boston, Massachusetts MA 02115, USA; 13 Harvard University, Cambridge, Massachusetts MA 02138, USA; 14 Data Archiving and Networked Services (DANS), The Hague 2593 HW, The Netherlands; 15 GigaScience, Beijing Genomics Institute, Shenzhen 518083, China; 16 Department of Bioinformatics, Maastricht University, Maastricht 6200 MD, The Netherlands; 17 Wageningen UR Plant Breeding, Wageningen 6708 PB, The Netherlands; 18 Oxford e-Research Center, University of Oxford, Oxford OX1 3QG, UK; 19 Heriot-Watt University, Edinburgh EH14 4AS, UK; 20 School of Computer Science, University of Manchester, Manchester M13 9PL, UK; 21 Center for Research in Biological Systems, School of Medicine, University of California San Diego, La Jolla, California 92093-0446, USA; 22 Dutch Techcenter for the Life Sciences, Utrecht 3501 DE, The Netherlands; 23 Department of Human Genetics, Leiden University Medical Center, Dutch Techcenter for the Life Sciences, Leiden 2300 RC, The Netherlands; 24 Dutch TechCenter for Life Sciences and ELIXIR-NL, Utrecht 3501 DE, The Netherlands; 25 VU University Amsterdam, Amsterdam 1081 HV, The Netherlands; 26 Leiden Center of Data Science, Leiden University, Leiden 2300 RA, The Netherlands; 27 Netherlands eScience Center, Amsterdam 1098 XG, The Netherlands; 28 National Center for Microscopy and Imaging Research, UCSD, San Diego 92103, USA; 29 Phortos Consultants, San Diego 92011, USA; 30 SciELO/FAPESP Program, UNIFESP Foundation, São Paulo 05468-901, Brazil; 31 Bioinformatics Infrastructure for Life Sciences (BILS), Science for Life Laboratory, Dept of Cell and Molecular Biology, Uppsala University, S-751 24, Uppsala, Sweden; 32 Leiden University Medical Center, Leiden 2333 ZA, The Netherlands; 33 Bayer CropScience, Gent Area 1831, Belgium; 34 Leiden Institute for Advanced Computer Science, Leiden University Medical Center, Leiden 2300 RA, The Netherlands; 35 Swiss Institute of Bioinformatics and University of Basel, Basel 4056, Switzerland; 36 Cray, Inc., Seattle 98164, USA; 37 Unaffiliated; 38 University Medical Center Groningen (UMCG), University of Groningen, Groningen 9713 GZ, The Netherlands; 39 Erasmus MC, Rotterdam 3015 CE, The Netherlands; 40 Independent Open Access and Open Science Advocate, Guildford GU1 3PW, UK; 41 Micelio, Antwerp 2180, Belgium; 42 Max Planck Compute and Data Facility, MPS, Garching 85748, Germany; 43 Leiden Institute of Advanced Computer Science, Leiden University, Leiden 2333 CA, The Netherlands; 44 Department of Computer Science, Oxford University, Oxford OX1 3QD, UK; 45 Leiden University Medical Center, Leiden and Dutch TechCenter for Life Sciences, Utrecht 2333 ZA, The Netherlands; 46 Netherlands eScience Center, Amsterdam 1098 XG, The Netherlands; 47 Erasmus MC, Rotterdam 3015 CE, The Netherlands

**Keywords:** Research data, Publication characteristics

## Abstract

There is an urgent need to improve the infrastructure supporting the reuse of scholarly data. A diverse set of stakeholders—representing academia, industry, funding agencies, and scholarly publishers—have come together to design and jointly endorse a concise and measureable set of principles that we refer to as the FAIR Data Principles. The intent is that these may act as a guideline for those wishing to enhance the reusability of their data holdings. Distinct from peer initiatives that focus on the human scholar, the FAIR Principles put specific emphasis on enhancing the ability of machines to automatically find and use the data, in addition to supporting its reuse by individuals. This Comment is the first formal publication of the FAIR Principles, and includes the rationale behind them, and some exemplar implementations in the community.

## Comment

### Supporting discovery through good data management

Good data management is not a goal in itself, but rather is the key conduit leading to knowledge discovery and innovation, and to subsequent data and knowledge integration and reuse by the community after the data publication process. Unfortunately, the existing digital ecosystem surrounding scholarly data publication prevents us from extracting maximum benefit from our research investments (e.g., ref. 1). Partially in response to this, science funders, publishers and governmental agencies are beginning to require data management and stewardship plans for data generated in publicly funded experiments. Beyond proper collection, annotation, and archival, data stewardship includes the notion of ‘long-term care’ of valuable digital assets, with the goal that they should be discovered and re-used for downstream investigations, either alone, or in combination with newly generated data. The outcomes from good data management and stewardship, therefore, are high quality digital publications that facilitate and simplify this ongoing process of discovery, evaluation, and reuse in downstream studies. What constitutes ‘good data management’ is, however, largely undefined, and is generally left as a decision for the data or repository owner. Therefore, bringing some clarity around the goals and desiderata of good data management and stewardship, and defining simple guideposts to inform those who publish and/or preserve scholarly data, would be of great utility.

This article describes four foundational principles—Findability, Accessibility, Interoperability, and Reusability—that serve to guide data producers and publishers as they navigate around these obstacles, thereby helping to maximize the added-value gained by contemporary, formal scholarly digital publishing. Importantly, it is our intent that the principles apply not only to ‘data’ in the conventional sense, but also to the algorithms, tools, and workflows that led to that data. All scholarly digital research objects^2^—from data to analytical pipelines—benefit from application of these principles, since all components of the research process must be available to ensure transparency, reproducibility, and reusability.

There are numerous and diverse stakeholders who stand to benefit from overcoming these obstacles: researchers wanting to share, get credit, and reuse each other’s data and interpretations; professional data publishers offering their services; software and tool-builders providing data analysis and processing services such as reusable workflows; funding agencies (private and public) increasingly concerned with long-term data stewardship; and a data science community mining, integrating and analysing new and existing data to advance discovery. To facilitate the reading of this manuscript by these diverse stakeholders, we provide definitions for common abbreviations in Box 1. Humans, however, are not the only critical stakeholders in the milieu of scientific data. Similar problems are encountered by the applications and computational agents that we task to undertake data retrieval and analysis on our behalf. These ‘computational stakeholders’ are increasingly relevant, and demand as much, or more, attention as their importance grows. One of the grand challenges of data-intensive science, therefore, is to improve knowledge discovery through assisting both humans, and their computational agents, in the discovery of, access to, and integration and analysis of, task-appropriate scientific data and other scholarly digital objects.

For certain types of important digital objects, there are well-curated, deeply-integrated, special-purpose repositories such as Genbank^3^, Worldwide Protein Data Bank (wwPDB^4^), and UniProt^5^ in the life sciences; Space Physics Data Facility (SPDF; http://spdf.gsfc.nasa.gov/) and Set of Identifications, Measurements and Bibliography for Astronomical Data (SIMBAD^6^) in the space sciences. These foundational and critical core resources are continuously curating and capturing high-value reference datasets and fine-tuning them to enhance scholarly output, provide support for both human and mechanical users, and provide extensive tooling to access their content in rich, dynamic ways. However, not all datasets or even data types can be captured by, or submitted to, these repositories. Many important datasets emerging from traditional, low-throughput bench science don’t fit in the data models of these special-purpose repositories, yet these datasets are no less important with respect to integrative research, reproducibility, and reuse in general. Apparently in response to this, we see the emergence of numerous general-purpose data repositories, at scales ranging from institutional (for example, a single university), to open globally-scoped repositories such as Dataverse^7^, FigShare (http://figshare.com), Dryad^8^, Mendeley Data (https://data.mendeley.com/), Zenodo (http://zenodo.org/), DataHub (http://datahub.io), DANS (http://www.dans.knaw.nl/), and EUDat^9^. Such repositories accept a wide range of data types in a wide variety of formats, generally do not attempt to integrate or harmonize the deposited data, and place few restrictions (or requirements) on the descriptors of the data deposition. The resulting data ecosystem, therefore, appears to be moving away from centralization, is becoming more diverse, and less integrated, thereby exacerbating the discovery and re-usability problem for both human and computational stakeholders.

A specific example of these obstacles could be imagined in the domain of gene regulation and expression analysis. Suppose a researcher has generated a dataset of differentially-selected polyadenylation sites in a non-model pathogenic organism grown under a variety of environmental conditions that stimulate its pathogenic state. The researcher is interested in comparing the alternatively-polyadenylated genes in this local dataset, to other examples of alternative-polyadenylation, and the expression levels of these genes—both in this organism and related model organisms—during the infection process. Given that there is no special-purpose archive for differential polyadenylation data, and no model organism database for this pathogen, where does the researcher begin?

We will consider the current approach to this problem from a variety of data discovery and integration perspectives. If the desired datasets existed, where might they have been published, and how would one begin to search for them, using what search tools? The desired search would need to filter based on specific species, specific tissues, specific types of data (Poly-A, microarray, NGS), specific conditions (infection), and specific genes—is that information (‘metadata’) captured by the repositories, and if so, what formats is it in, is it searchable, and how? Once the data is discovered, can it be downloaded? In what format(s)? Can that format be easily integrated with private in-house data (the local dataset of alternative polyadenylation sites) as well as other data publications from third-parties and with the community’s core gene/protein data repositories? Can this integration be done automatically to save time and avoid copy/paste errors? Does the researcher have permission to use the data from these third-party researchers, under what license conditions, and who should be cited if a data-point is re-used?

Questions such as these highlight some of the barriers to data discovery and reuse, not only for humans, but even more so for machines; yet it is precisely these kinds of deeply and broadly integrative analyses that constitute the bulk of contemporary e-Science. The reason that we often need several weeks (or months) of specialist technical effort to gather the data necessary to answer such research questions is not the lack of appropriate technology; the reason is, that we do not pay our valuable digital objects the careful attention they deserve when we create and preserve them. Overcoming these barriers, therefore, necessitates that all stakeholders—including researchers, special-purpose, and general-purpose repositories—evolve to meet the emergent challenges described above. The goal is for scholarly digital objects of all kinds to become ‘first class citizens’ in the scientific publication ecosystem, where the quality of the publication—and more importantly, the impact of the publication—is a function of its ability to be accurately and appropriately found, re-used, and cited over time, by all stakeholders, both human and mechanical.

With this goal in-mind, a workshop was held in Leiden, Netherlands, in 2014, named ‘Jointly Designing a Data Fairport’. This workshop brought together a wide group of academic and private stakeholders all of whom had an interest in overcoming data discovery and reuse obstacles. From the deliberations at the workshop the notion emerged that, through the definition of, and widespread support for, a minimal set of community-agreed guiding principles and practices, all stakeholders could more easily discover, access, appropriately integrate and re-use, and adequately cite, the vast quantities of information being generated by contemporary data-intensive science. The meeting concluded with a draft formulation of a set of foundational principles that were subsequently elaborated in greater detail—namely, that all research objects should be Findable, Accessible, Interoperable and Reusable (FAIR) both for machines and for people. These are now referred to as the FAIR Guiding Principles. Subsequently, a dedicated FAIR working group, established by several members of the FORCE11 community^10^ fine-tuned and improved the Principles. The results of these efforts are reported here.

### The significance of machines in data-rich research environments

The emphasis placed on FAIRness being applied to both human-driven and machine-driven activities, is a specific focus of the FAIR Guiding Principles that distinguishes them from many peer initiatives (discussed in the subsequent section). Humans and machines often face distinct barriers when attempting to find and process data on the Web. Humans have an intuitive sense of ‘semantics’ (the meaning or intent of a digital object) because we are capable of identifying and interpreting a wide variety of contextual cues, whether those take the form of structural/visual/iconic cues in the layout of a Web page, or the content of narrative notes. As such, we are less likely to make errors in the selection of appropriate data or other digital objects, although humans will face similar difficulties if sufficient contextual metadata is lacking. The primary limitation of humans, however, is that we are unable to operate at the scope, scale, and speed necessitated by the scale of contemporary scientific data and complexity of e-Science. It is for this reason that humans increasingly rely on computational agents to undertake discovery and integration tasks on their behalf. This necessitates machines to be capable of autonomously and appropriately acting when faced with the wide range of types, formats, and access-mechanisms/protocols that will be encountered during their self-guided exploration of the global data ecosystem. It also necessitates that the machines keep an exquisite record of provenance such that the data they are collecting can be accurately and adequately cited. Assisting these agents, therefore, is a critical consideration for all participants in the data management and stewardship process—from researchers and data producers to data repository hosts.

Throughout this paper, we use the phrase ‘machine actionable’ to indicate a continuum of possible states wherein a digital object provides increasingly more detailed information to an autonomously-acting, computational data explorer. This information enables the agent—to a degree dependent on the amount of detail provided—to have the capacity, when faced with a digital object never encountered before, to: a) identify the type of object (with respect to both structure and intent), b) determine if it is useful within the context of the agent’s current task by interrogating metadata and/or data elements, c) determine if it is usable, with respect to license, consent, or other accessibility or use constraints, and d) take appropriate action, in much the same manner that a human would.

For example, a machine may be capable of determining the data-type of a discovered digital object, but not capable of parsing it due to it being in an unknown format; or it may be capable of processing the contained data, but not capable of determining the licensing requirements related to the retrieval and/or use of that data. The optimal state—where machines fully ‘understand’ and can autonomously and correctly operate-on a digital object—may rarely be achieved. Nevertheless, the FAIR principles provide ‘steps along a path’ toward machine-actionability; adopting, in whole or in part, the FAIR principles, leads the resource along the continuum towards this optimal state. In addition, the idea of being machine-actionable applies in two contexts—first, when referring to the contextual metadata surrounding a digital object (‘what is it?’), and second, when referring to the content of the digital object itself (‘how do I process it/integrate it?’). Either, or both of these may be machine-actionable, and each forms its own continuum of actionability.

Finally, we wish to draw a distinction between data that is machine-actionable as a result of specific investment in software supporting that data-type, for example, bespoke parsers that understand life science wwPDB files or space science Space Physics Archive Search and Extract (SPASE) files, and data that is machine-actionable exclusively through the utilization of general-purpose, open technologies. To reiterate the earlier point—ultimate machine-actionability occurs when a machine can make a useful decision regarding data that it has not encountered before. This distinction is important when considering both (a) the rapidly growing and evolving data environment, with new technologies and new, more complex data-types continuously being developed, and (b) the growth of general-purpose repositories, where the data-types likely to be encountered by an agent are unpredictable. Creating bespoke parsers, in all computer languages, for all data-types and all analytical tools that require those data-types, is not a sustainable activity. As such, the focus on assisting machines in their discovery and exploration of data through application of more generalized interoperability technologies and standards at the data/repository level, becomes a first-priority for good data stewardship.

### The FAIR Guiding Principles in detail

Representatives of the interested stakeholder-groups, discussed above, coalesced around four core desiderata—the FAIR Guiding Principles—and limited elaboration of these, which have been refined (Box 2) from the meeting’s original draft, available at (https://www.force11.org/node/6062). A separate document that dynamically addresses community discussion relating to clarifications and explanations of the principles, and detailed guidelines for and examples of FAIR implementations, is currently being constructed (http://datafairport.org/fair-principles-living-document-menu). The FAIR Guiding Principles describe distinct considerations for contemporary data publishing environments with respect to supporting both manual and automated deposition, exploration, sharing, and reuse. While there have been a number of recent, often domain-focused publications advocating for specific improvements in practices relating to data management and archival^1,11,12^, FAIR differs in that it describes concise, domain-independent, high-level principles that can be applied to a wide range of scholarly outputs. Throughout the Principles, we use the phrase ‘(meta)data’ in cases where the Principle should be applied to both metadata and data.

The elements of the FAIR Principles are related, but independent and separable. The Principles define characteristics that contemporary data resources, tools, vocabularies and infrastructures should exhibit to assist discovery and reuse by third-parties. By minimally defining each guiding principle, the barrier-to-entry for data producers, publishers and stewards who wish to make their data holdings FAIR is purposely maintained as low as possible. The Principles may be adhered to in any combination and incrementally, as data providers’ publishing environments evolve to increasing degrees of ‘FAIRness’. Moreover, the modularity of the Principles, and their distinction between data and metadata, explicitly support a wide range of special circumstances. One such example is highly sensitive or personally-identifiable data, where publication of rich metadata to facilitate discovery, including clear rules regarding the process for accessing the data, provides a high degree of ‘FAIRness’ even in the absence of FAIR publication of the data itself. A second example involves the publication of non-data research objects. Analytical workflows, for example, are a critical component of the scholarly ecosystem, and their formal publication is necessary to achieve both transparency and scientific reproducibility. The FAIR principles can equally be applied to these non-data assets, which need to be identified, described, discovered, and reused in much the same manner as data.

Specific exemplar efforts that provide varying levels of FAIRness are detailed later in this document. Additional issues, however, remain to be addressed. First, when community-endorsed vocabularies or other (meta)data standards do not include the attributes necessary to achieve rich annotation, there are two possible solutions: either publish an extension of an existing, closely related vocabulary, or—in the extreme case—create and explicitly publish a new vocabulary resource, following FAIR principles (‘I2’). Second, to explicitly identify the standard chosen when more than one vocabulary or other (meta)data standard is available, and given that for instance in the life sciences there are over 600 content standards, the BioSharing registry (https://biosharing.org/) can be of use as it describes the standards in detail, including versions where applicable.

### The Principles precede implementation

These high-level FAIR Guiding Principles precede implementation choices, and do not suggest any specific technology, standard, or implementation-solution; moreover, the Principles are not, themselves, a standard or a specification. They act as a guide to data publishers and stewards to assist them in evaluating whether their particular implementation choices are rendering their digital research artefacts Findable, Accessible, Interoperable, and Reusable. We anticipate that these high level principles will enable a broad range of integrative and exploratory behaviours, based on a wide range of technology choices and implementations. Indeed, many repositories are already implementing various aspects of FAIR using a variety of technology choices and several examples are detailed in the next section; examples include *Scientific Data* itself and how narrative data articles are anchored to a progressively FAIR structured metadata.

### Examples of FAIRness, and the resulting value-added

**Dataverse**^7^: Dataverse is an open-source data repository software installed in dozens of institutions globally to support public community repositories or institutional research data repositories. Harvard Dataverse, with more than 60,000 datasets, is the largest of the current Dataverse repositories, and is open to all researchers from all research fields. Dataverse generates a formal citation for each deposit, following the standard defined by Altman and King^13^. Dataverse makes the Digital Object Identifier (DOI), or other persistent identifiers (Handles), public when the dataset is published (‘F’). This resolves to a landing page, providing access to metadata, data files, dataset terms, waivers or licenses, and version information, all of which is indexed and searchable (‘F’, ‘A’, and ‘R’). Deposits include metadata, data files, and any complementary files (such as documentation or code) needed to understand the data and analysis (‘R’). Metadata is always public, even if the data are restricted or removed for privacy issues (‘F’, ‘A’). This metadata is offered at three levels, extensively supporting the ‘I’ and ‘R’ FAIR principles: 1) data citation metadata, which maps to DataCite schema or Dublin Core Terms, 2) domain-specific metadata, which when possible maps to metadata standards used within a scientific domain, and 3) file-level metadata, which can be deep and extensive for tabular data files (including column-level metadata). Finally, Dataverse provides public machine-accessible interfaces to search the data, access the metadata and download the data files, using a token to grant access when data files are restricted (‘A’).

**FAIRDOM** (http://fair-dom.org/about): integrates the SEEK^14^ and openBIS^15^ platforms to produce a FAIR data and model management facility for Systems Biology. Individual research assets (or aggregates of data and models) are identified with unique and persistent HTTP URLs, which can be registered with DOIs for publication (‘F’). Assets can be accessed over the Web in a variety of formats appropriate for individuals and/or their computers (RDF, XML) (‘I’). Research assets are annotated with rich metadata, using community standards, formats and ontologies (‘I’). The metadata is stored as RDF to enable interoperability and assets can be downloaded for reuse (‘R’).

**ISA**^16^: is a community-driven metadata tracking framework to facilitate standards-compliant collection, curation, management and reuse of life science datasets. ISA provides progressively FAIR structured metadata to Nature Scientific Data’s Data Descriptor articles, and many GigaScience data papers, and underpins the EBI MetaboLights database among other data resources. At the heart is a general-purpose, extensible ISA model, originally only available as a tabular representation but subsequently enhanced as an RDF-based representation^17^, and JSON serializations to enable the ‘I’ and ‘R’, becoming ‘FAIR’ when published as linked data (http://elixir-uk.org/node-events/201cisa-as-a-fair-research-object201d-hack-the-spec-event-1) and complementing other research objects^18^.

**Open PHACTS**^19^: Open PHACTS is a data integration platform for information pertaining to drug discovery. Access to the platform is mediated through a machine-accessible interface^20^ which provides multiple representations that are both human (HTML) and machine readable (RDF, JSON, XML, CSV, etc), providing the ‘A’ facet of FAIRness. The interface allows multiple URLs to be used to access information about a particular entity through a mappings service (‘F’ and ‘A’). Thus, a user can provide a ChEMBL URL to retrieve information sourced from, for example, Chemspider or DrugBank. Each call provides a canonical URL in its response (‘A’ and ‘I’). All data sources used are described using standardized dataset descriptions, following the global VoID standard, with rich provenance (‘R’ and ‘I’). All interface features are described using RDF following the Linked Data API specification (‘A’). Finally, a majority of the datasets are described using community agreed upon ontologies (‘I’).

**wwPDB**^4,21^: wwPDB is a special-purpose, intensively-curated data archive that hosts information about experimentally-determined 3D structures of proteins and nucleic acids. All wwPDB entries are stably hosted on an FTP server (‘A’) and represented in machine-readable formats (text and XML); the latter are machine-actionable using the metadata provided by the wwPDB conforming to the Macromolecular Information Framework (mmCIF^22^), a data standard of the International Union of Crystallography (IUCr) (‘F’,‘I’ for humans, ‘F’,‘I’ for IUCr-aware machines). The wwPDB metadata contains cross-references to common identifiers such as PubMed and NCBI Taxonomy, and their wwPDB metadata are described in data dictionaries and schema documents (http://mmcif.wwpdb.org and http://pdbml.wwpdb.org) which conform to the IUCr data standard for the chemical and structural biology domains (‘R’). A variety of software tools are available to interpret both wwPDB data and meta-data (‘I’,‘R’ for humans, ‘I’,‘R’ for machines with this software). Each entry is represented by a DOI (‘F’, ‘A’ for humans and machines). The DOI resolves to a zipped file which requires special software for further interrogation/interpretation. Other wwPDB access points^23–25^ provide access to wwPDB records through URLs that are likely to be stable in the long-term (‘F’), and all data and metadata is searchable through one or more of the wwPDB-affiliated websites (‘F’)

**UniProt**^26^: UniProt is a comprehensive resource for protein sequence and annotation data. All entries are uniquely identified by a stable URL, that provides access to the record in a variety of formats including a web page, plain-text, and RDF (‘F’ and ‘A’). The record contains rich metadata (‘F’) that is both human-readable (HTML) and machine-readable (text and RDF), where the RDF formatted response utilizes shared vocabularies and ontologies such as UniProt Core, FALDO, and ECO (‘I’). Interlinking with more than 150 different databases, every UniProt record has extensive links into, for example, PubMed, enabling rich citation. These links are machine-actionable in the RDF representation (‘R’). Finally, in the RDF representation, the UniProt Core Ontology explicitly types all records, leaving no ambiguity—neither for humans nor machines—about what the data represents (‘R’), enabling fully-automated retrieval of records and cross-referencing information.

In addition to, and in support of, communities and resources that are already pursuing FAIR objectives, the Data Citation Implementation Group of Force11 has published specific technical recommendations for how to implement many of the principles^27^, with a particular focus on identifiers and their resolution, persistence, and metadata accessibility especially related to citation. In addition, the ‘Skunkworks’ group that emerged from the Lorentz Workshop has been creating software supporting infrastructures^28^ that are, end-to-end, compatible with FAIR principles, and can be implemented over existing repositories. These code modules have a particular focus on metadata publication and searchability, compatibility in cases of strict privacy considerations, and the extremely difficult problem of data and metadata interoperability (manuscript in preparation). Finally, there are several emergent projects, some listed in Box 3, for which FAIR is a key objective. These projects may provide valuable advice and guidance for those wishing to become more FAIR.

### FAIRness is a prerequisite for proper data management and data stewardship

The ideas within the FAIR Guiding Principles reflect, combine, build upon and extend previous work by both the Concept Web Alliance (https://conceptweblog.wordpress.com/) partners, who focused on machine-actionability and harmonization of data structures and semantics, and by the scientific and scholarly organizations that developed the Joint Declaration of Data Citation Principles (JDDCP^29^), who focused on primary scholarly data being made citable, discoverable and available for reuse, so as to be capable of supporting more rigorous scholarship. An attempt to define the similarities and overlaps between the FAIR Principles and the JDDCP is provided at (https://www.force11.org/node/6062). The FAIR Principles are also complementary to the ‘Data Seal of Approval’ (DSA) (http://datasealofapproval.org/media/filer_public/2013/09/27/guidelines_2014-2015.pdf) in that they share the general aim to render data re-usable for users other than those who originally generated them. While the DSA focuses primarily on the responsibilities and conduct of data producers and repositories, FAIR focuses primarily on the data itself. Clearly, the broader community of stakeholders is coalescing around a set of common, dovetailed visions spanning all facets of the scholarly data publishing ecosystem.

The end result, when implemented, will be more rigorous management and stewardship of these valuable digital resources, to the benefit of the entire academic community. As stated at the outset, good data management and stewardship is not a goal in itself, but rather a pre-condition supporting knowledge discovery and innovation. Contemporary e-Science requires data to be Findable, Accessible, Interoperable, and Reusable in the long-term, and these objectives are rapidly becoming expectations of agencies and publishers. We demonstrate, therefore, that the FAIR Data Principles provide a set of mileposts for data producers and publishers. They guide the implementation of the most basic levels of good Data Management and Stewardship practice, thus helping researchers adhere to the expectations and requirements of their funding agencies. We call on all data producers and publishers to examine and implement these principles, and actively participate with the FAIR initiative by joining the Force11 working group. By working together towards shared, common goals, the valuable data produced by our community will gradually achieve the critical goals of FAIRness.

## Additional Information

**How to cite this article:** Wilkinson, M. D. *et al.* The FAIR Guiding Principles for scientific data management and stewardship. *Sci. Data* 3:160018 doi: 10.1038/sdata.2016.18 (2016).

## Terms and Abbreviations

**BD2K**—Big Data 2 Knowledge, is a trans-NIH initiative established to enable biomedical research as a digital research enterprise, to facilitate discovery and support new knowledge, and to maximise community engagement.

**DOI**—Digital Object Identifier; a code used to permanently and stably identify (usually digital) objects. DOIs provide a standard mechanism for retrieval of metadata about the object, and generally a means to access the data object itself.

**FAIR**—Findable, Accessible, Interoperable, Reusable.

**FORCE11**—The Future of Research Communications and e-Scholarship; a community of scholars, librarians, archivists, publishers and research funders that has arisen organically to help facilitate the change toward improved knowledge creation and sharing, initiated in 2011.

**Interoperability**—the ability of data or tools from non-cooperating resources to integrate or work together with minimal effort.

**JDDCP**—Joint Declaration of Data Citation Principles; Acknowledging data as a first-class research output, and to support good research practices around data re-use, JDDCP proposes a set of guiding principles for citation of data within scholarly literature, another dataset, or any other research object.

**RDF**—Resource Description Framework; a globally-accepted framework for data and knowledge representation that is intended to be read and interpreted by machines.

## The FAIR Guiding Principles

**To be Findable:**

F1. (meta)data are assigned a globally unique and persistent identifier

F2. data are described with rich metadata (defined by R1 below)

F3. metadata clearly and explicitly include the identifier of the data it describes

F4. (meta)data are registered or indexed in a searchable resource

**To be Accessible:**

A1. (meta)data are retrievable by their identifier using a standardized communications protocol

A1.1 the protocol is open, free, and universally implementable

A1.2 the protocol allows for an authentication and authorization procedure, where necessary

A2. metadata are accessible, even when the data are no longer available

**To be Interoperable:**

I1. (meta)data use a formal, accessible, shared, and broadly applicable language for knowledge representation.

I2. (meta)data use vocabularies that follow FAIR principles

I3. (meta)data include qualified references to other (meta)data

**To be Reusable:**

R1. meta(data) are richly described with a plurality of accurate and relevant attributes

R1.1. (meta)data are released with a clear and accessible data usage license

R1.2. (meta)data are associated with detailed provenance

R1.3. (meta)data meet domain-relevant community standards

## Emergent community/collaborative initiatives with FAIR as a core focus or activity

**bioCADDIE** (https://biocaddie.org): The NIH BD2K biomedical and healthCAre Data Discovery Index Ecosystem (bioCADDIE) consortium works to develop a Data Discovery Index (DDI) prototype, which is set to be as transformative and impactful for data as PubMed for the biomedical literature^30^. The DDI focuses on finding (‘F’) and accessing (‘A’) the datasets stored across different sources, and progressively works to identify relevant metadata^31^ (‘I’) and maps them to community standards (‘R’), linking to BioSharing.

**CEDAR**^32^: The Center for Expanded Data Annotation and Retrieval (CEDAR) is an NIH BD2K funded center of excellence to develop tools and technologies that reduce the burden of authoring and enhancing metadata that meet community-based standards. CEDAR will enable the creation of metadata templates that implement community based standards for experimental metadata, from BioSharing (https://biosharing.org), and that will be uniquely identifiable and retrievable with HTTP URIs, and annotated with vocabularies and ontologies drawn from BioPortal (http://bioportal.bioontology.org) (‘F’,‘A’,‘I’,‘R’). These templates will guide users to create rich metadata with unique and stable HTTP identifiers (‘F’) that can be retrieved using HTTP (‘A’) and accessible in a variety of formats (JSON-LD, TURTLE, RDF/XML, CSV, etc) (‘I’). These metadata will use community standards, as defined by the template, and include provenance and data usage (‘R’).

These two projects, among others, provide tools and or collaborative opportunities for those who wish to improve the FAIRness of their data.
