# Addendum: The FAIR Guiding Principles for scientific data management and stewardship

**DOI:** 10.1038/s41597-019-0009-6

**Published:** 2019-03-19

**Authors:** Mark D. Wilkinson, Michel Dumontier, Ijsbrand Jan Aalbersberg, Gabrielle Appleton, Myles Axton, Arie Baak, Niklas Blomberg, Jan-Willem Boiten, Luiz Bonino da Silva Santos, Philip E. Bourne, Jildau Bouwman, Anthony J. Brookes, Tim Clark, Mercè Crosas, Ingrid Dillo, Olivier Dumon, Scott Edmunds, Chris T. Evelo, Richard Finkers, Alejandra Gonzalez-Beltran, Alasdair J. G. Gray, Paul Groth, Carole Goble, Jeffrey S. Grethe, Jaap Heringa, Peter A. C. ’t Hoen, Rob Hooft, Tobias Kuhn, Ruben Kok, Joost Kok, Scott J. Lusher, Maryann E. Martone, Albert Mons, Abel L. Packer, Bengt Persson, Philippe Rocca-Serra, Marco Roos, Rene van Schaik, Susanna-Assunta Sansone, Erik Schultes, Thierry Sengstag, Ted Slater, George Strawn, Morris A. Swertz, Mark Thompson, Johan van der Lei, Erik van Mulligen, Jan Velterop, Andra Waagmeester, Peter Wittenburg, Katherine Wolstencroft, Jun Zhao, Barend Mons

**Affiliations:** 1 Center for Plant Biotechnology and Genomics, Universidad Politécnica de Madrid, Madrid, 28223 Spain; 2 Stanford University, Stanford, 94305-5411 USA; 3 Elsevier, Amsterdam, 1043 NX The Netherlands; 4 Nature Genetics, New York, 10004-1562 USA; 5 Euretos and Phortos Consultants, Rotterdam, 2741 CA The Netherlands; 6 ELIXIR, Wellcome Genome Campus, Hinxton, CB10 1SA UK; 7 Lygature, Eindhoven, 5656 AG The Netherlands; 8 Vrije Universiteit Amsterdam, Dutch Techcenter for Life Sciences, Amsterdam, 1081 HV The Netherlands; 9 Office of the Director, National Institutes of Health, Rockville, 20892 USA; 10 TNO, Zeist, 3700 AJ The Netherlands; 11 Department of Genetics, University of Leicester, Leicester, LE1 7RH UK; 12 Harvard Medical School, Boston, Massachusetts, MA 02115 USA; 13 Harvard University, Cambridge, Massachusetts, MA 02138 USA; 14 Data Archiving and Networked Services (DANS), The Hague, 2593 HW The Netherlands; 15 GigaScience, Beijing Genomics Institute, Shenzhen, 518083 China; 16 Department of Bioinformatics, Maastricht University, Maastricht, 6200 MD The Netherlands; 17 Wageningen UR Plant Breeding, Wageningen, 6708 PB The Netherlands; 18 Oxford e-Research Center, University of Oxford, Oxford, OX1 3QG UK; 19 Heriot-Watt University, Edinburgh, EH14 4AS UK; 20 School of Computer Science, University of Manchester, Manchester, M13 9PL UK; 21 Center for Research in Biological Systems, School of Medicine, University of California San Diego, La Jolla, California, 92093-0446 USA; 22 Dutch Techcenter for the Life Sciences, Utrecht, 3501 DE The Netherlands; 23 Department of Human Genetics, Leiden University Medical Center, Dutch Techcenter for the Life Sciences, Leiden, 2300 RC The Netherlands; 24 Dutch TechCenter for Life Sciences and ELIXIR-NL, Utrecht, 3501 DE The Netherlands; 25 VU University Amsterdam, Amsterdam, 1081 HV The Netherlands; 26 Leiden Center of Data Science, Leiden University, Leiden, 2300 RA The Netherlands; 27 Netherlands eScience Center, Amsterdam, 1098 XG The Netherlands; 28 National Center for Microscopy and Imaging Research, UCSD, San Diego, 92103 USA; 29 Phortos Consultants, San Diego, 92011 USA; 30 SciELO/FAPESP Program, UNIFESP Foundation, São Paulo, 05468-901 Brazil; 31 Bioinformatics Infrastructure for Life Sciences (BILS), Science for Life Laboratory, Dept of Cell and Molecular Biology, Uppsala University, S-751 24 Uppsala, Sweden; 32 Leiden University Medical Center, Leiden, 2333 ZA The Netherlands; 33 Bayer CropScience, Gent Area, 1831 Belgium; 34 Leiden Institute for Advanced Computer Science, Leiden University Medical Center, Leiden, 2300 RA The Netherlands; 35 Swiss Institute of Bioinformatics and University of Basel, Basel, 4056 Switzerland; 36 Cray, Inc., Seattle, 98164 USA; 37 Unaffiliated, Washington, D.C. USA; 38 University Medical Center Groningen (UMCG), University of Groningen, Groningen, 9713 GZ The Netherlands; 39 Erasmus MC, Rotterdam, 3015 CE The Netherlands; 40 Independent Open Access and Open Science Advocate, Guildford, GU1 3PW UK; 41 Micelio, Antwerp, 2180 Belgium; 42 Max Planck Compute and Data Facility, MPS, Garching, 85748 Germany; 43 Leiden Institute of Advanced Computer Science, Leiden University, Leiden, 2333 CA The Netherlands; 44 Department of Computer Science, Oxford University, Oxford, OX1 3QD UK; 45 Leiden University Medical Center, Leiden and Dutch TechCenter for Life Sciences, Utrecht, 2333 ZA The Netherlands; 46 Netherlands eScience Center, Amsterdam, 1098 XG The Netherlands; 47 Erasmus MC, Rotterdam, 3015 CE The Netherlands

Addendum to: *Scientific Data* 10.1038/sdata.2016.18, published online 15 March 2016

Since publication, the URL at which the FAIR principles ‘living document’ is hosted and maintained has changed from http://datafairport.org/fair-principles-living-document-menu to https://www.go-fair.org/fair-principles/.

